# Supplementary material for: Structure and mechanism of the K+/H+ exchanger KefC
Source: Nat Commun. 2024 Jun 4;15:4751. doi: 10.1038/s41467-024-49082-7 (PMC11150392; doi:10.1038/s41467-024-49082-7)
Supplement: Supplementary file 1 — Supplementary Information [file 41467_2024_49082_MOESM1_ESM.pdf]

Supplementary information

Structure and mechanism of the K^+/H^+ exchanger KefC

Ashutosh Gulati[1*], Surabhi Kokane[1*], Annemarie Perez Boerema[1], Claudia Alleva[1], Pascal F. Meier[1], Rei Matsuoka[1], David Drew[1#]

[1]Department of Biochemistry and Biophysics, Science for Life Laboratory, Stockholm University, SE–106 91 Stockholm, Sweden.

***These authors contributed equally**

#Correspondence: ddrew@dbb.su.se

This file contains Supplementary Figures 1-14 and Supplementary Table 1

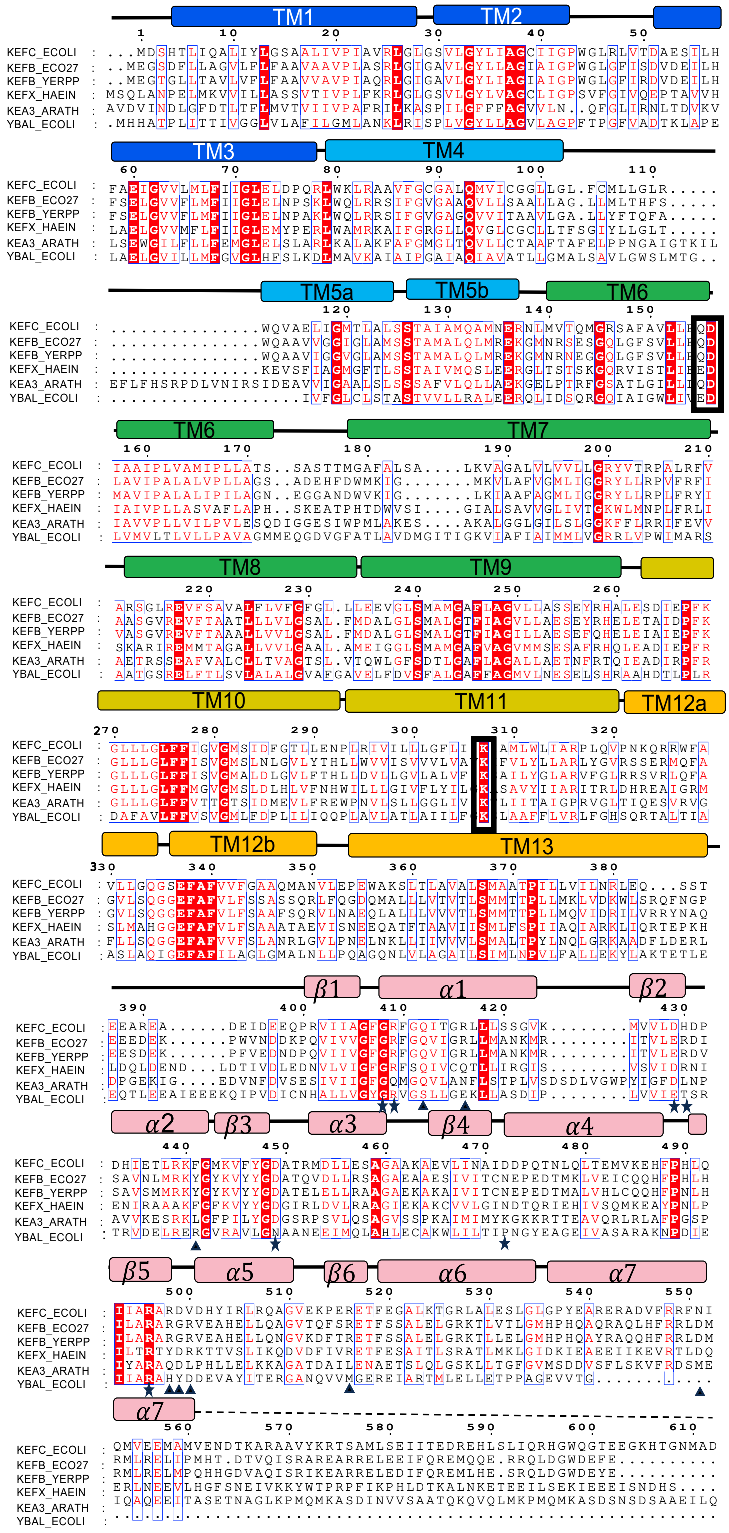

Supplementary Fig. 1. Multiple sequence alignment for KefC and its homologs. KefC *E. coli* (P03819) aligns with KefB *E. coli* (B7UK61) with 42% sequence identity, KefB *Yersinia pestis* (A4TGX5) with 41% sequence identity, KefX *Haemophilus influenzae*

(P44933) with 35.2% sequence identity, KEA3 *Arabidopsis thaliana* (Q9M0Z3) with 23.8% sequence identity and Ybal *E. coli* (P39830) with 22.7% sequence identity, respectively. Residues with over 90% sequence identity are indicated by red background. Active site residues are highlighted in black box. Residues which interact with AMP are marked with star and those binding GSH are indicated with triangle**.**

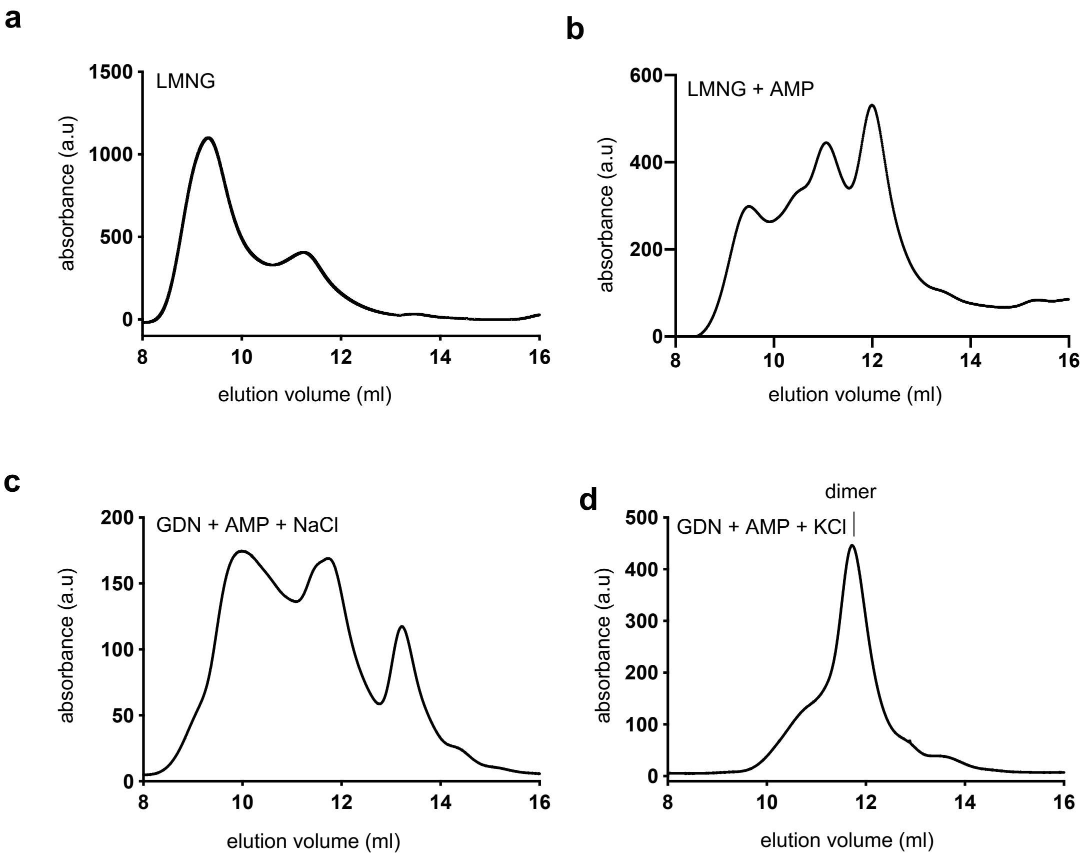

Supplementary Fig. 2. Optimization of KefC purification. **a.** Size-exclusion chromatography (SEC) profile of KefC WT* purified in Tris-HCl pH 7.5 supplemented with 150 mM NaCl and the detergent LMNG. The peak at 8.5 ml corresponds to aggregated protein **b.** As in a., but with the nucleotide AMP added in the purification buffer. **c.** As in b., but with the detergent GDN. **d.** As in c., but with the NaCl salt exchanged for KCl.

a

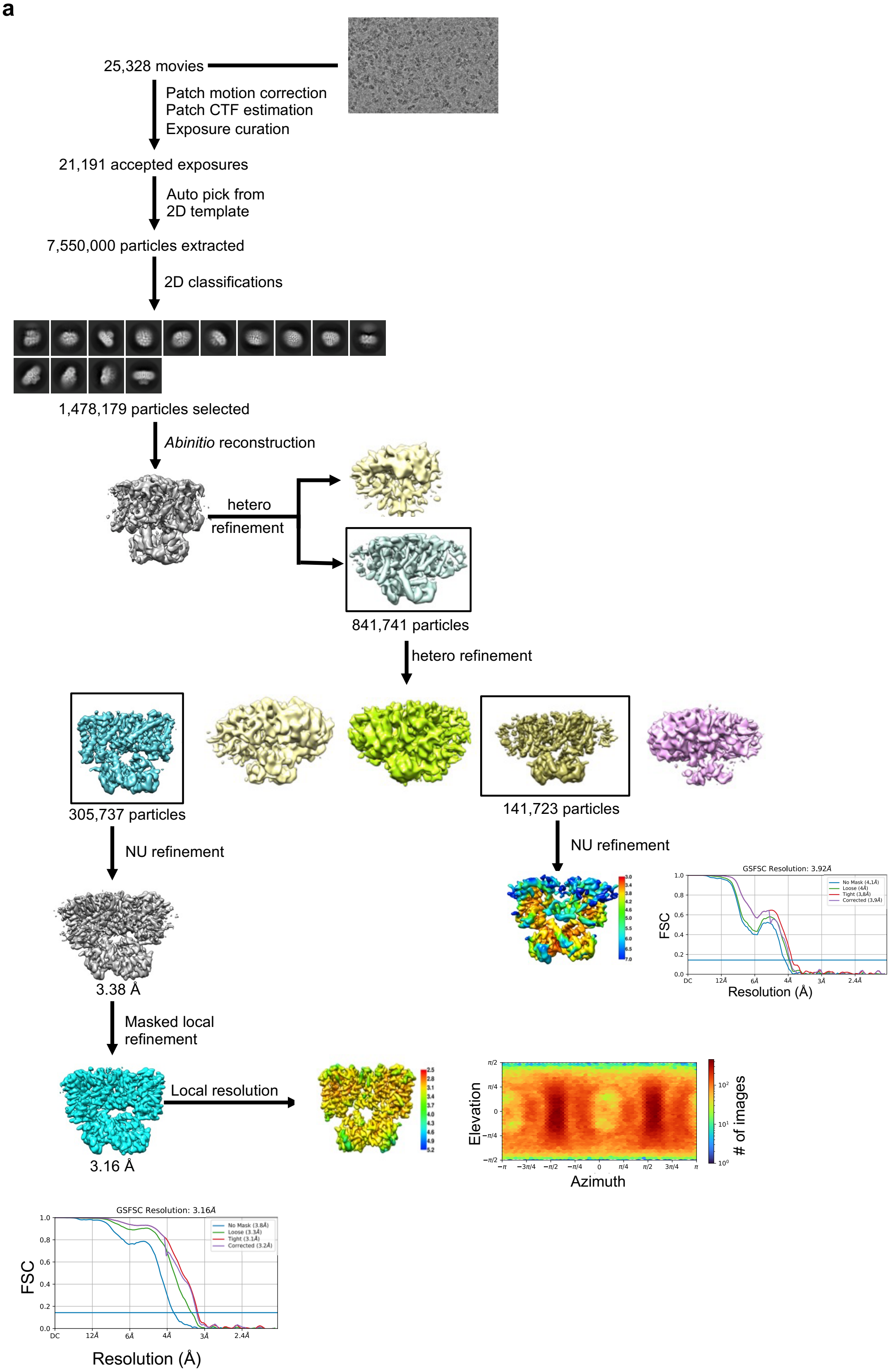

25,328 movies
Patch motion correction
Patch CTF estimation
Exposure curation
21,191 accepted exposures
Auto pick from
2D template
7,550,000 particles extracted
2D classifications
1,478,179 particles selected
Abinitio reconstruction
hetero
refinement
841,741 particles
hetero refinement
305,737 particles
141,723 particles
NU refinement
NU refinement
GSFSC Resolution: 3.92Å
FSC
Resolution (Å)
3.38 Å
Masked local
refinement
Local resolution
3.16 Å
Elevation
Azimuth
# of images
GSFSC Resolution: 3.16Å
FSC
Resolution (Å)

b

Supplementary Fig. 3. The data-processing workflow of KefC. a. The dataset contained 25,328 movies that were corrected by Patch motion correction and Patch CTF estimation in CryoSPARC[1]. After reference-based auto-picking, 7,550,000 particles were picked. Several rounds of 2D classification were performed, yielding 1,478,149 particles, which were subjected to *ab initio* reconstructions and heterogenous refinements. One of the 3D classes was selected, and it contained 841,741 particles. After repeated heterogenous refinement a further two main classes were selected with one class achieving a final resolution of 3.16 Å at gold-standard FSC (0.143), with a local resolution range of 2.7–4.5 Å. **b.** The FSC curve for the final KefC homodimer in the detergent GDN.

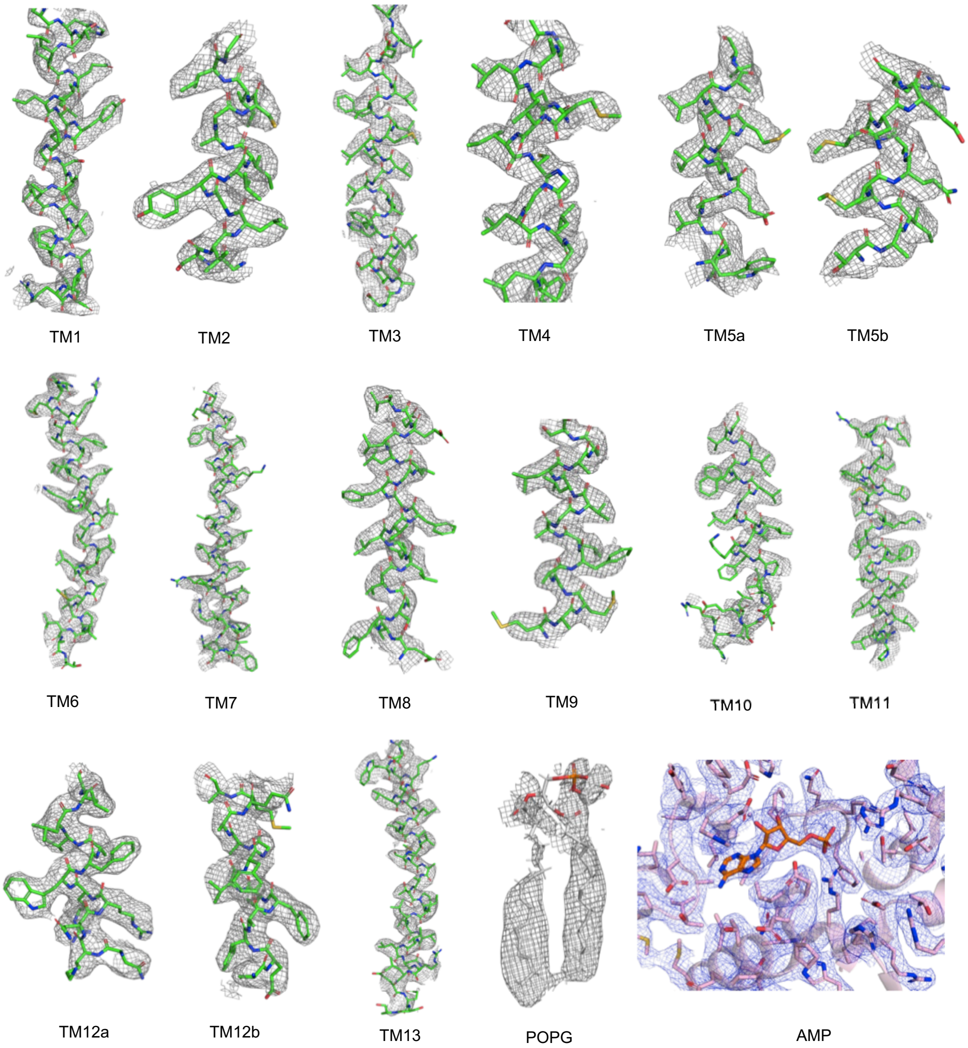

Supplementary Fig. 4. Cryo-EM density of KefC. a. Cryo-EM density map and model are shown for all the transmembrane segments for KefC and AMP, that fitted into both nucleotide-binding pockets of the RCK domains.

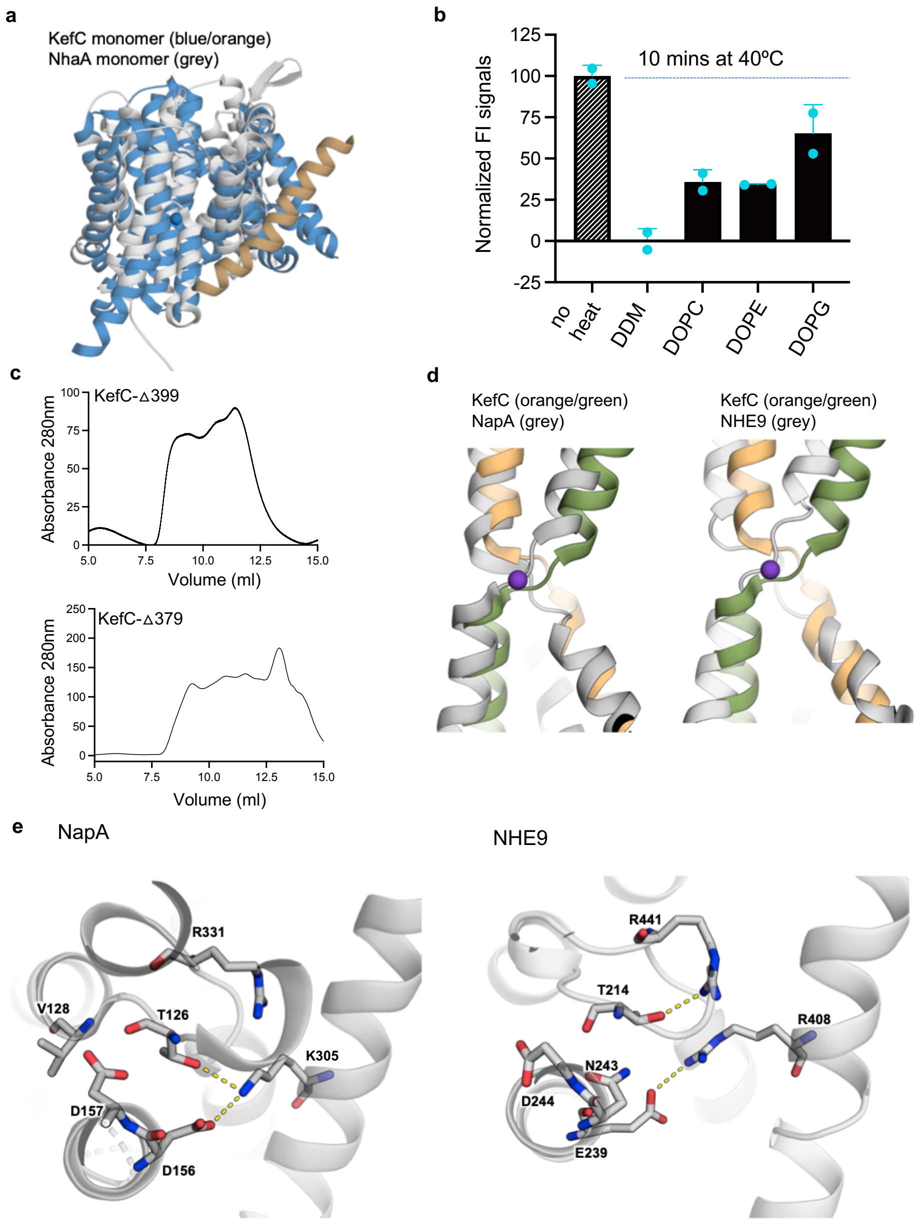

Supplementary Fig. 5. Structural comparison between KefC and bacterial Na^{+}/H^{+} exchangers. a. Superimposition of 13-TM KefC (blue and TM1 in orange) with the NhaA monomer (grey) in an inward-facing conformation (PDB: 4ATV). **b.** Thermal stabilization of DDM-purified dimeric KefC WT*-GFP by lipids. Normalized mean fluorescence (r.f.u., relative fluorescence units) is shown after heating (40 °C, for 10 mins), centrifugation and FSEC in the presence of either the detergent DDM or DDM-solubilized lipids. Error bars represent the range of n = 2 independent experiments (Methods). The source data is provided in source data file **c.** Size exculsion profiles for truncated versions of KefC (KefC-Δ399 and KefC-Δ379) indicating that KefC transporter

module is prone to aggregation in the absence of RCK domains. **d.** Superimposition of helical cross-overs of KefC (light orange and green) with Na^+/H^+ antiporters NapA (left, PDB: 5BZ2) and NHE9 (right, PDB: 6Z3Z) showing the longer helical breaks in NapA and NHE9 in comparison to KefC. **e**. Top-down view of the putative Na^+ binding site of NapA (left) and NHE9 (right) from the extracellular side. Neutralization of the TM12a-b negative dipoles by arginine (R331 and R441), which is a conserved feature in all 13-TM Na^+/H^+ exchangers is shown. The structurally equivalent residue is a Q334 in KefC, which is also highly conserved in other Kef homologs. Conserved positively charged residue of TM11, K305 of NapA and R408 of NHE9 interacts with residues present in TM6, D156 of NapA and E239 of NHE9, respectively. Equivalent K307 of TM11 in KefC instead interacts with residues from TM5 and TM12, thereby stabilizes the more compact K^+ binding site.

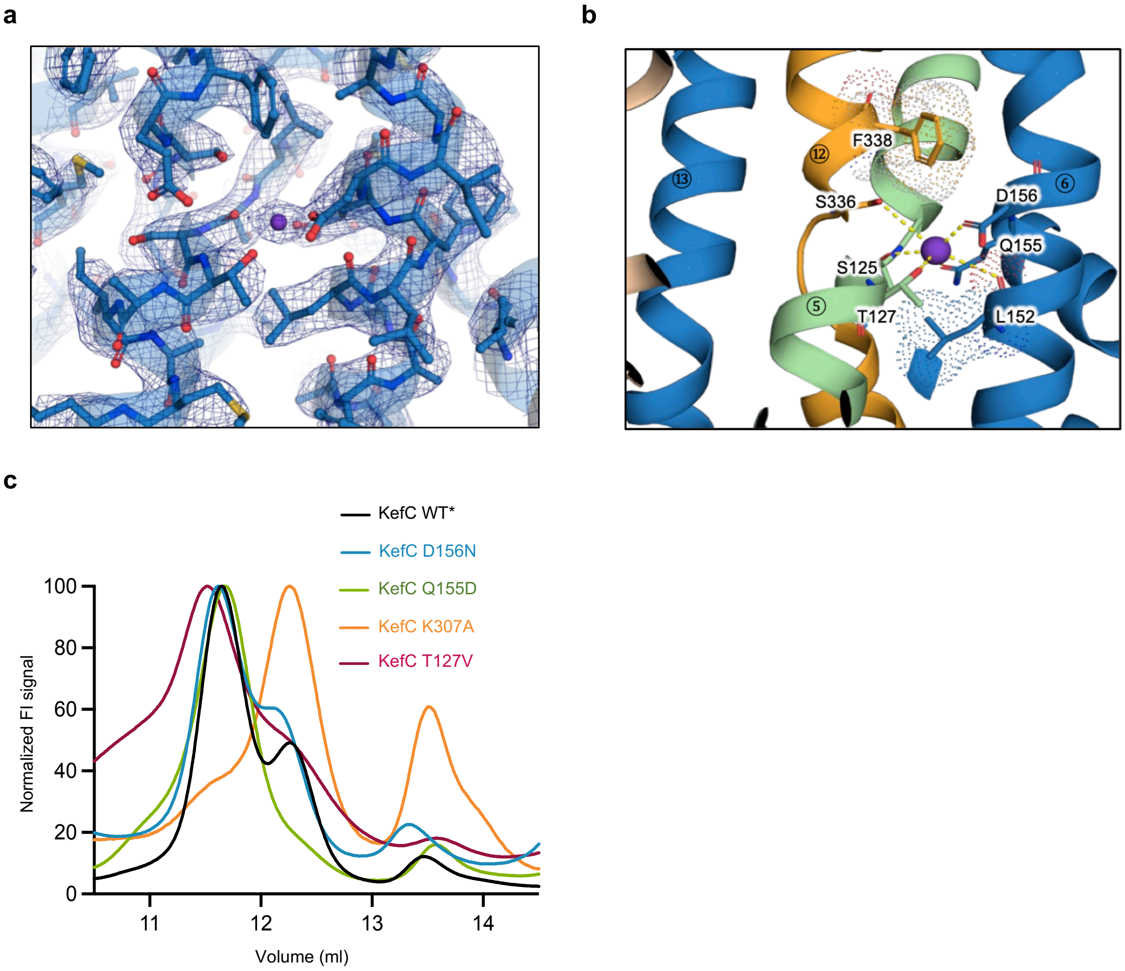

Supplementary Fig. 6. Characterization of ion coordination in KefC. a. Cryo-EM map density of K^+ binding site and its surrounding residues (12 Å radii) in the sharpened map of KefC AMP bound structure. Map density was contoured at 7.2σ in PyMol[2]. Similar ion density was also observed in the KefC AMP and GSH bound structure. **b.** The ion-binding site of KefC is gated by highly conserved hydrophobic residues F338 and L152, which is a common feature of Na^+/H^+ exchanger ion-binding sites[3,4]. **c.** Representative normalized FSEC traces of purified GFP fused KefC WT* (black) and K^+ binding site variants; D156N (blue), Q155D (green), K307A (orange), and T127V (magenta). Dimeric KefC could be observed in all variants except K307A, where, only monomeric protein could be observed.

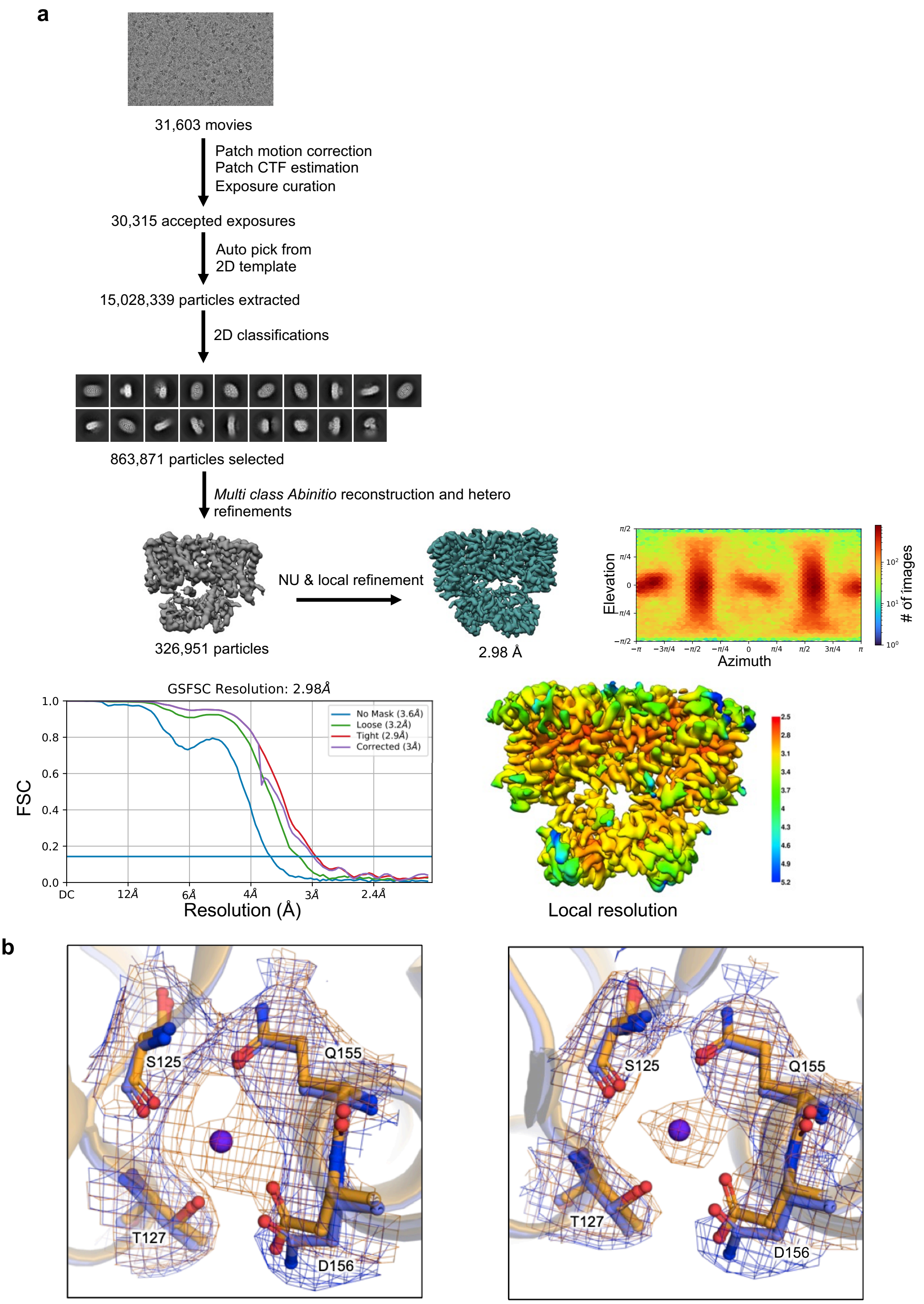

Supplementary Fig. 7. The data-processing workflow of KefC D156N variant a. The dataset contained 31,603 movies that were corrected by Patch motion correction and

Patch CTF estimation in CryoSPARC[1]. After reference-based auto-picking, 15,028,339 particles were picked. Several rounds of 2D classification were performed, yielding 863,871 particles, which were subjected to *ab initio* reconstructions and heterogenous refinements. One of the 3D classes was selected, and it contained 326,951 particles which was further refined to a final resolution of 2.98 Å at gold-standard FSC of 0.143. **b.** Map overlay of KefC WT* (orange) and D156N variant (blue), resampled and rendered at same thresholds showing the same high-quality of density for the side-chains (orange and blue sticks), no signal for the K^+ ion in the D156N cryo-EM maps were observed in both *left:* unsharpened map (left) and phenix autosharpened maps (right).

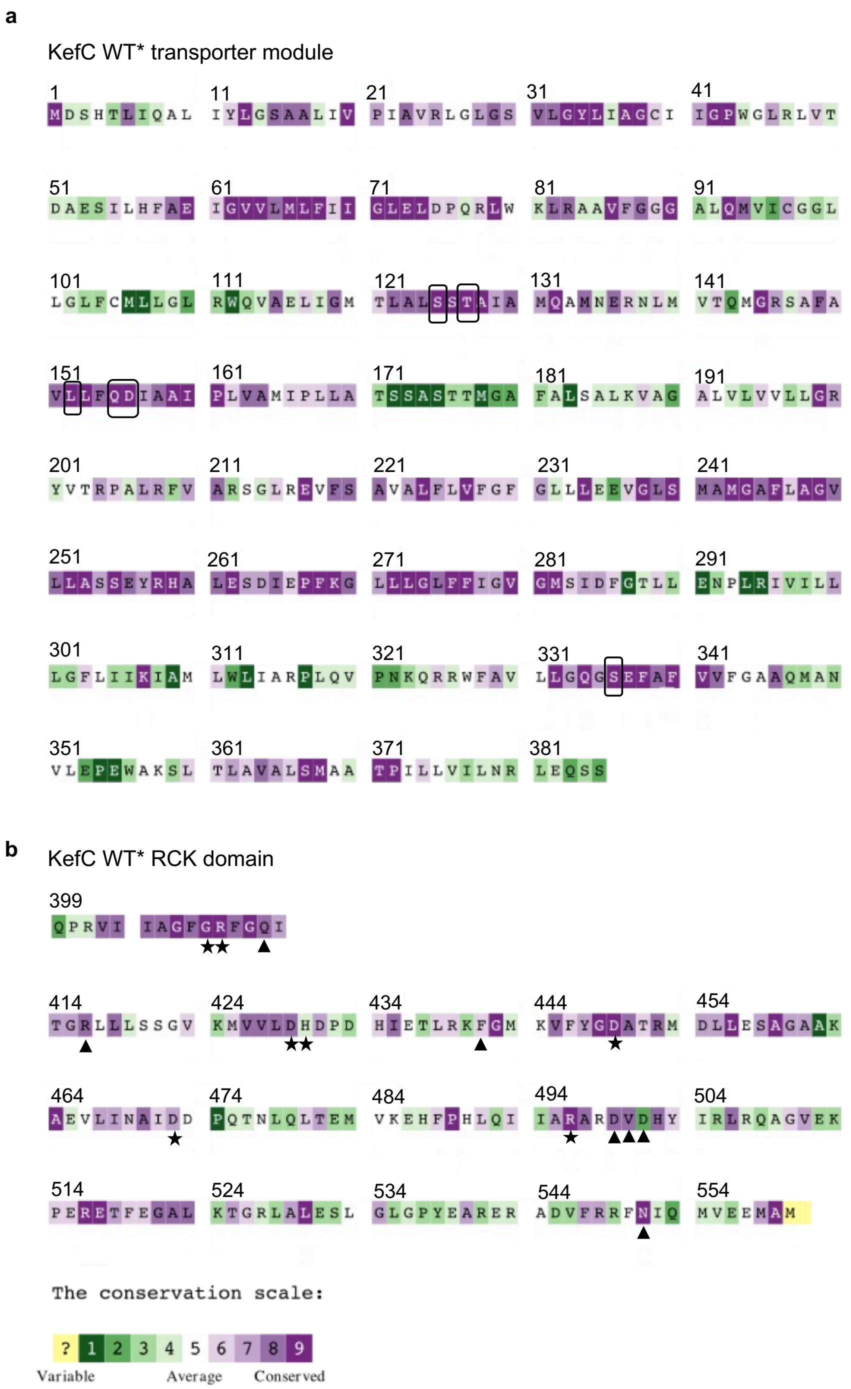

Supplementary Fig. 8. ConSurf analysis of *E. coli* KefC using default parameters of the ConSurf server[5]. **a.** Sequence conservation annotation for the transporter module. Residues important for K^+ binding are boxed. **b.** Sequence conservation annotation for the RCK domain. Residues important for AMP and GSH binding are marked with stars and triangles, respectively.

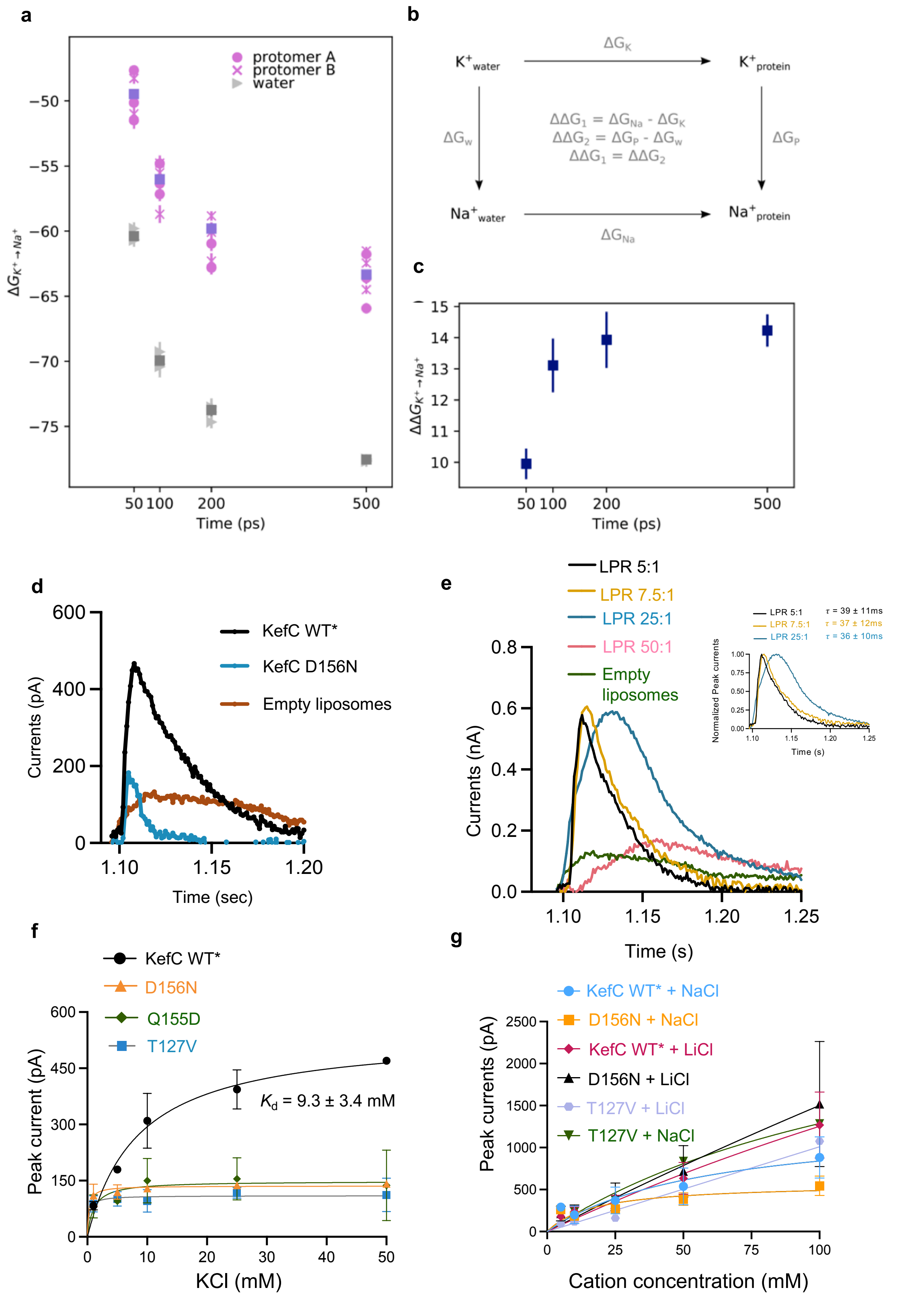

Supplementary Fig. 9. MD-simulation and SSM-based electrophysiology of KefC is consistent with an electroneutral transporter specific to K^+ ions. a Calculated ΔG for

the K^+ to Na^+ transition in protomer A (circles), protomer B (crosses) and water (triangles) for a switching time of 50 ps, 100 ps, 200 ps and 500 ps replicas are shown in lighter colors, while the mean is shown in darker colors and as squares. **b.** Thermodynamic cycle reporting the calculation for the $\Delta\Delta G$ of the K^+ to Na^+ transition. **c.** Calculated $\Delta\Delta G$ for the K^+ to Na^+ transition, as obtained by the difference between the ΔG of the transition in water and in the protein. **d.** Transient currents recorded on KefC proteoliposomes under symmetrical pH 7.8 and upon addition of 25 mM KCl to WT* (black trace), and KefC variant in which the ion-binding aspartate was substituted to asparagine (orange trace). **e.** Transient currents recorded under symmetrical pH 7.8 and upon addition of 25 mM KCl to KefC WT* proteoliposomes prepared with different LPR ratios 5:1(black), 7.5:1(yellow), 25:1 (blue), 50:1 (pink) and empty liposomes (green). Inset: The normalized peak currents for LPR 5:1, 7.5:1 and 25:1 is shown with their respective decay constants (**τ**). The decay constant was calculated using n = 3 titrations (independent sensors) **f.** Fit of the peak transient currents as a function of K^+ concentrations for KefC WT* and the corresponding binding affinity (K_d). No clear binding affinity could be calculated for the D156N (orange), Q155D (green) and T127V (blue) variants. Data are mean ± s.d. of $n = 3$ titrations (sensors) that were each measured in triplicate. **g.** Fit of the transient currents as a function of Li^+ and Na^+ concentrations for WT, D156N, and the T127V variant as annotated. Data are mean values ± s.d. of $n = 3$ individual titrations (sensors) that were each recorded in triplicate. No clear binding affinity for Na^+ or Li^+ could be calculated for any of the KefC construct. The source data for Supplementary fig 8e, 8f and 8g is provided in source data file.

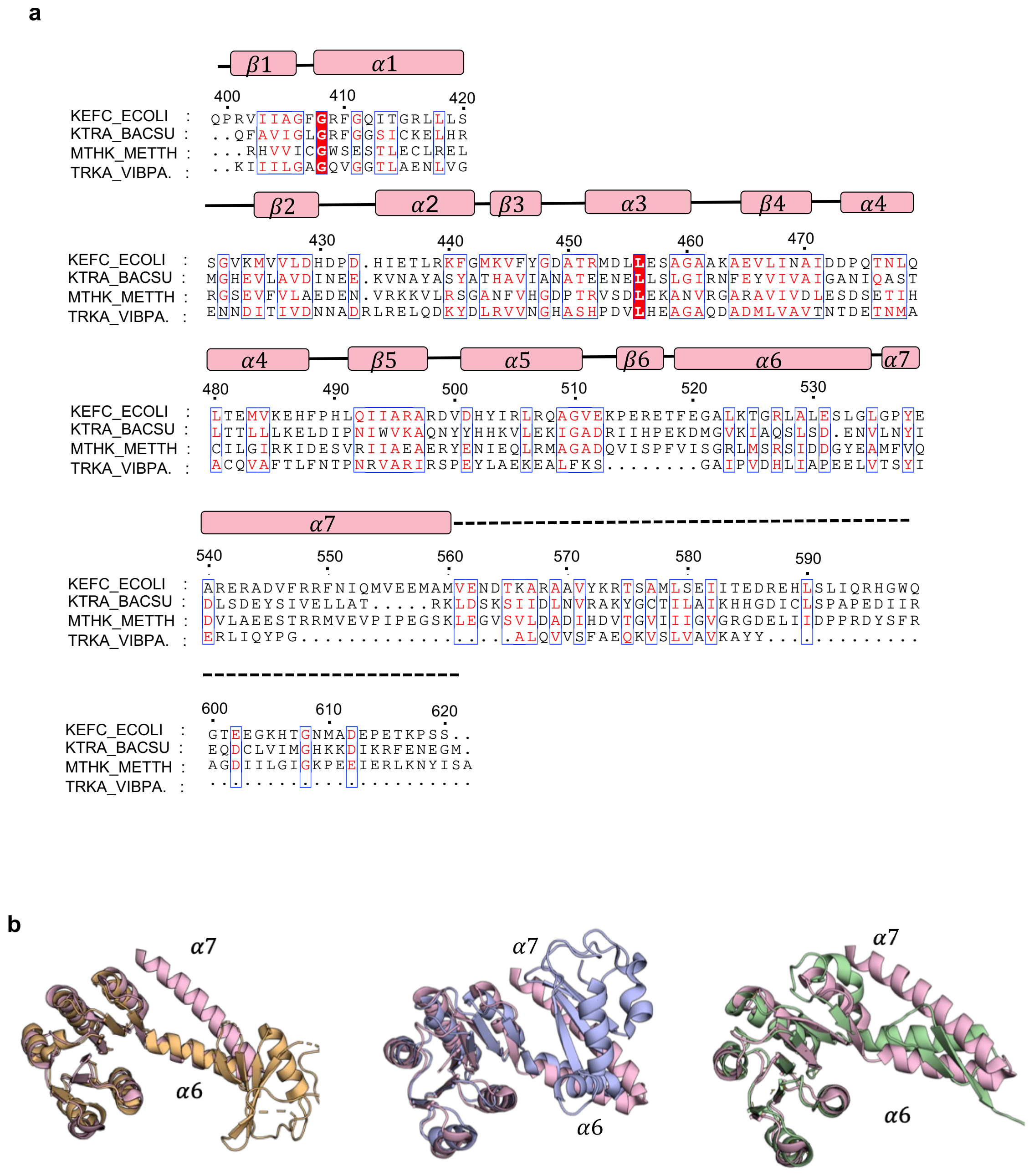

Supplementary Fig. 10. Comparison of KefC RCK domain with RCK domains from potassium channels a. Sequence alignment of KTN and RCK domains from KEFC_ECOLI (Accession Id: P03819) and KTRA_BASCU (Accession Id: O32080) with sequence identity of 17.89%, MTHK_METTH (Accession Id: O27564) with sequence identity of 21.65%, TRKA_VIBPA (Accession Id: Q87TN7) with sequence identity of 25%. **b.** Superimposition of RCK domains from KEFC_ECOLI (PDB:8BXG) (light pink) and KTRA_BASCU (PDB:4J91) (light orange) with rmsd of 1.4, MTHK_METTH (PDB:6OLY) (light blue) with rmsd of 2.4, TRKA_VIBPA (PDB:

4J9V) (pale green) with rmsd of 1.4. Helix $\alpha7$, which is unique to KefC RCK domain forms extensive contacts with the transport module.

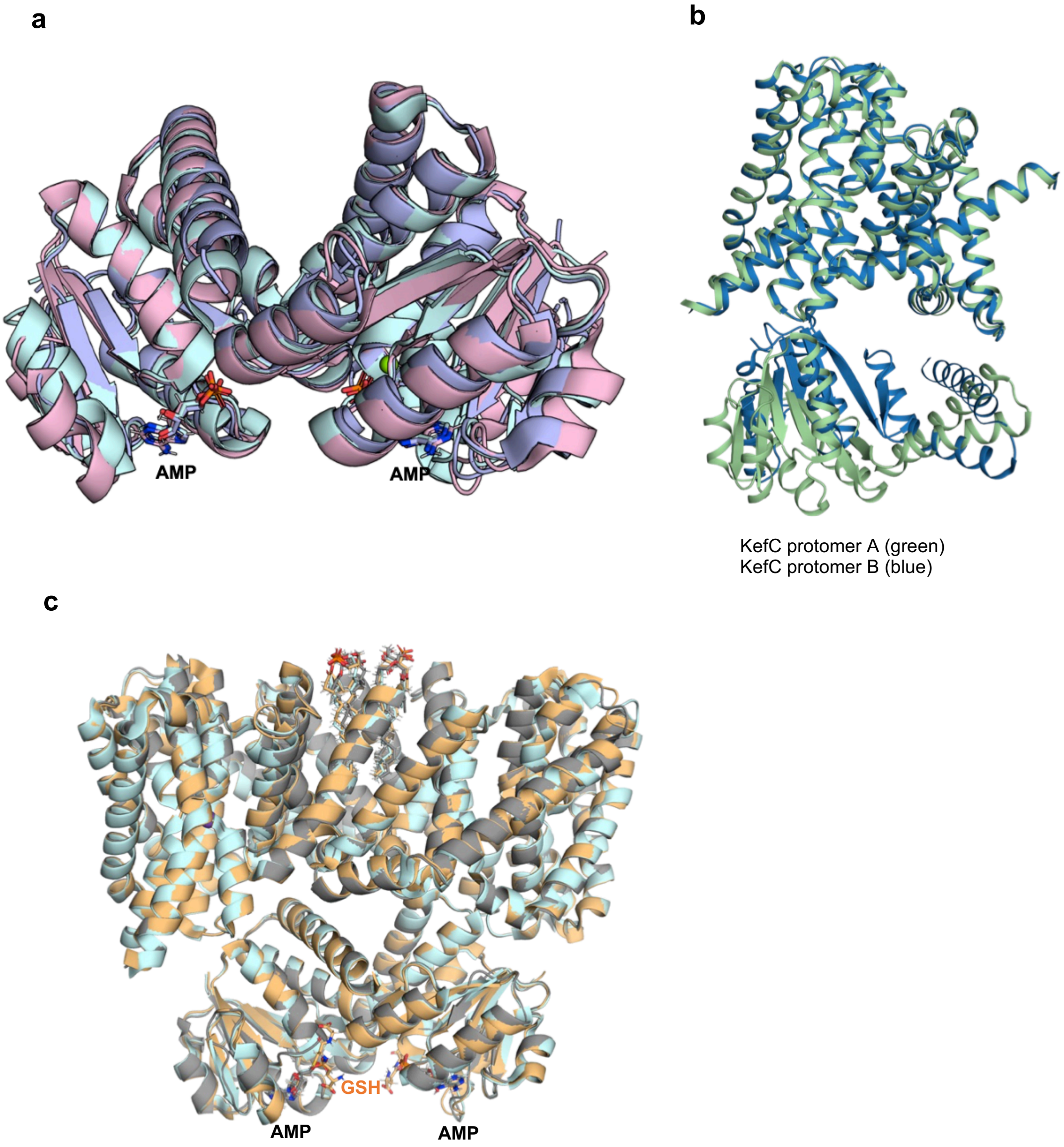

Supplementary Fig. 11. Asymmetric interaction of RCK dimer domain with the transporter module. a. Structural comparison of KefC RCK domains in the cryo-EM structure (pink) with the previously determined crystal structures of the soluble RCK-domain (PDB: 5NC8; blue and PDB: 3EYW; cyan). **b.** Superimposition of the KefC protomers highlighting the different positions of the RCK domains relative to the KefC transporter module. **c.** Superimposition of KefC WT*(cyan), D156N (grey) and KefC WT* with GSH (orange) with Cα rmsd of around 0.5 Å. Asymmmetric interactions between RCK domains and the transporter module is observed consistently in all the structures of KefC.

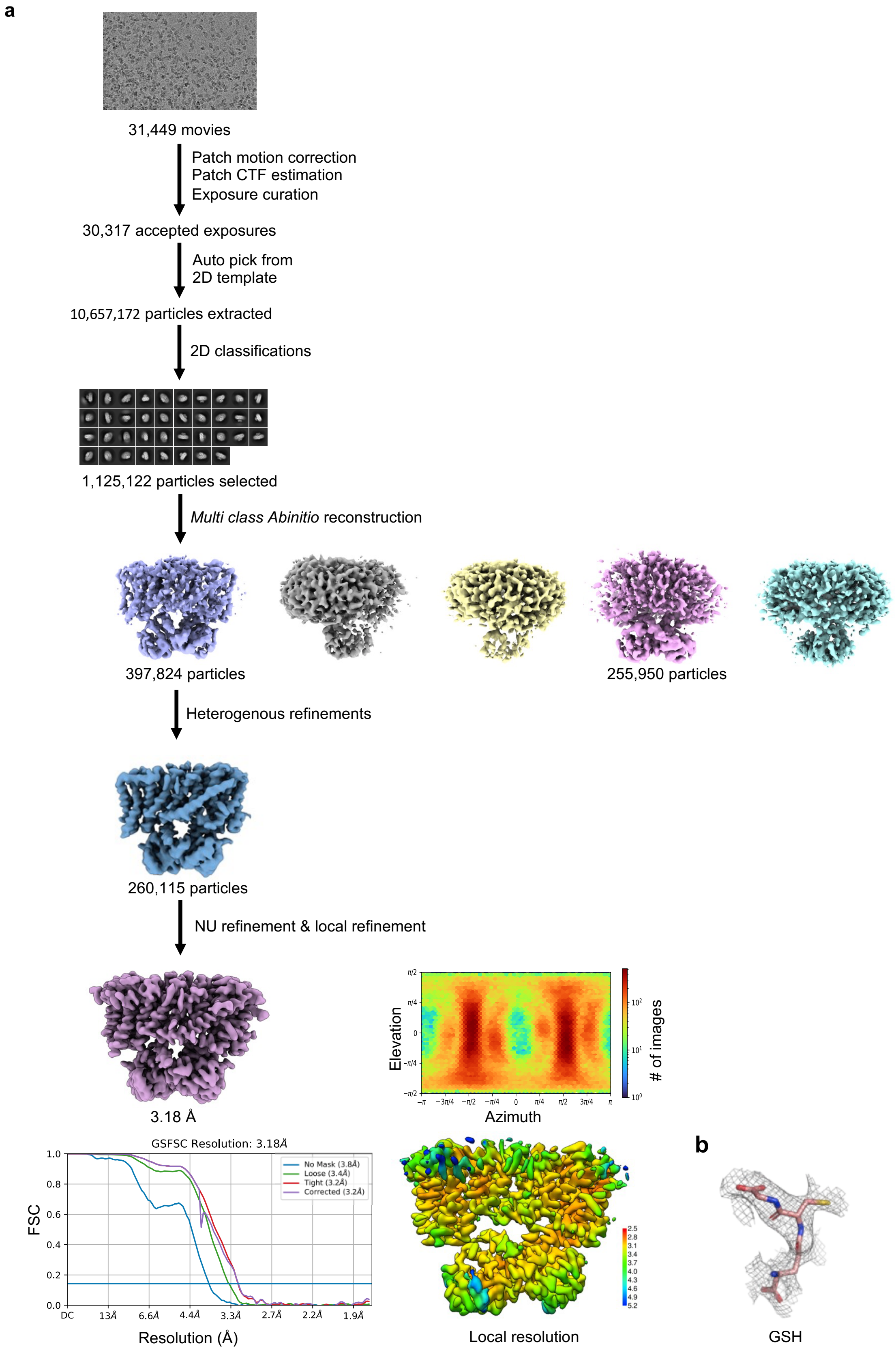
a
31,449 movies
Patch motion correction
Patch CTF estimation
Exposure curation
30,317 accepted exposures
Auto pick from
2D template
10,657,172 particles extracted
2D classifications
1,125,122 particles selected
Multi class Abinitio reconstruction
397,824 particles
255,950 particles
Heterogenous refinements
260,115 particles
NU refinement & local refinement
3.18 Å
Elevation
Azimuth
# of images
GSFSC Resolution: 3.18Å
No Mask (3.8Å)
Loose (3.4Å)
Tight (3.2Å)
Corrected (3.2Å)
FSC
Resolution (Å)
Local resolution
b
GSH

Supplementary Fig. 12. The data-processing workflow of KefC WT* with AMP and GSH. a. The dataset contained 31,449 movies that were corrected by Patch motion correction and Patch CTF estimation in CryoSPARC[1]. After reference-based auto-picking, 10,657,172 particles were picked. Several rounds of 2D classification were performed, yielding 1,125,122 particles, which were subjected to *ab initio* reconstructions and heterogenous refinements. One of the 3D classes was selected, and it contained 260,115 particles which was further refined to a final resolution of 3.18 Å at gold-standard FSC of 0.143. **b.** Cryo-EM map for GSH.

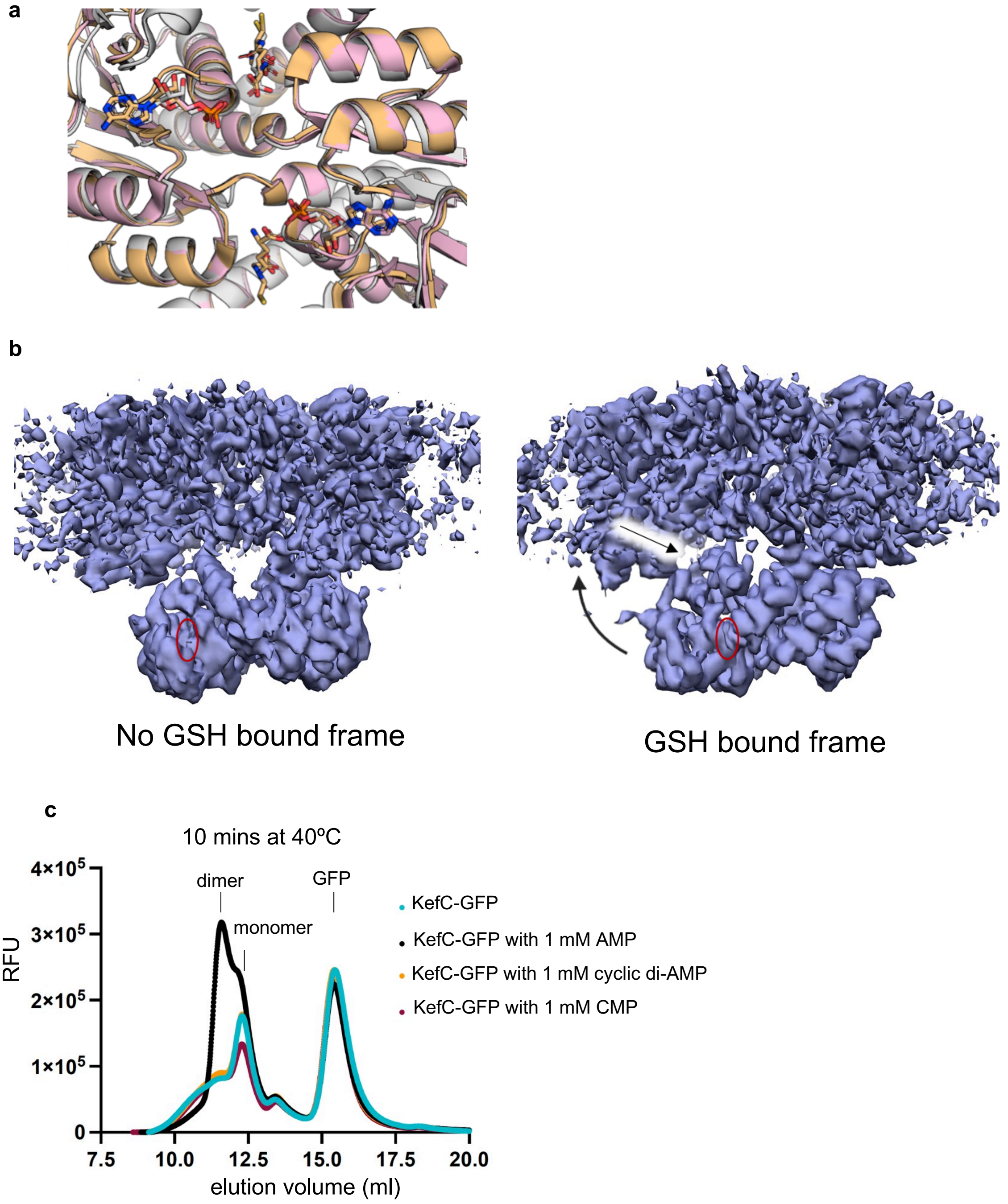

Supplementary Fig. 13. 3DVA of GSH-bound KefC WT* cryo-EM maps and 2D classes of KefC collection without AMP. a. Superimpositions of KefC cryo-EM structure with AMP only (pink)(PDB: 8BXG), AMP and GSH (sand)(PDB: 8BY2) and crystal structure of soluble RCK domain with AMP and GSH (PDB: 3L9W).**b**. A frame of the 3DVA of GSH and AMP bound KefC WT* structure with no clear map density for GSH (red oval). As in a, but for clearer density for GSH (red oval), which is correlated

with more contacts between the RCK domains and the KefC transporter module (black arrow): the frames in a-b, are taken from Supplementary Movie 1. **c**. Thermal stabilization of DDM solubilized membranes of KefC WT*-GFP by nucleotides (Methods). Heat FSEC traces of KefC WT*-GFP solubilized in DDM only (cyan trace), with 1 mM AMP (black trace), 1 mM cyclic di-AMP (yellow trace) and 1 mM CMP (burgundy trace).

a

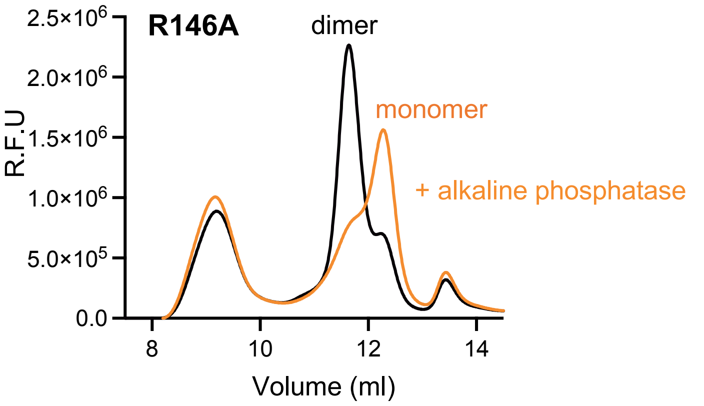

b

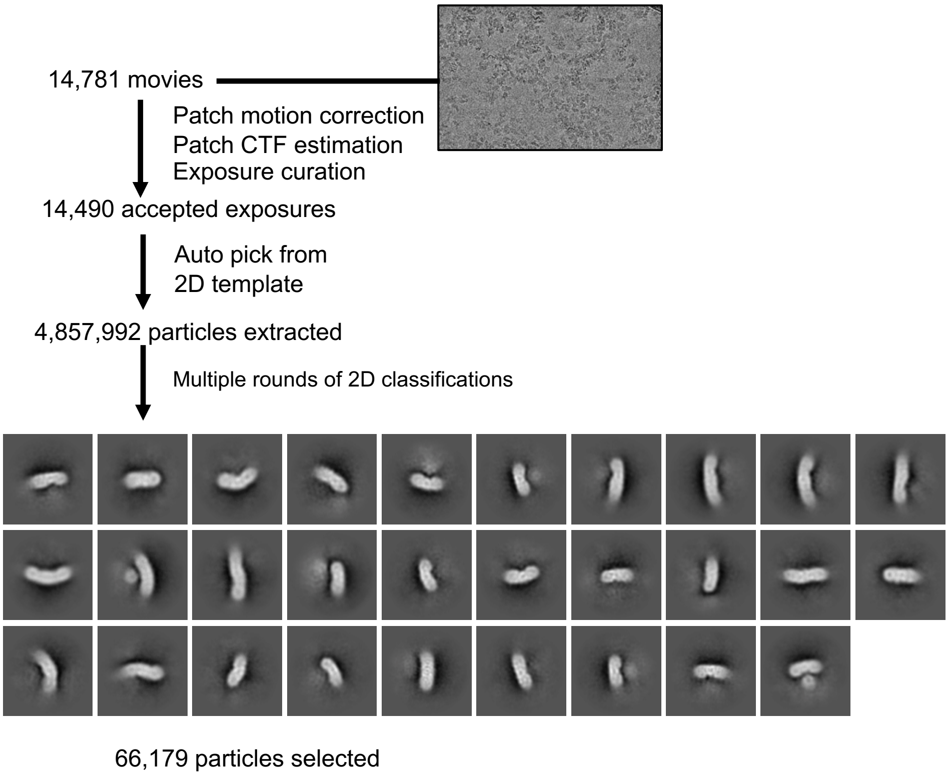

Supplementary Fig. 14. Removal of AMP leads to KefC dimer instability. a. FSEC traces of DDM solubilized membranes of KefC R146A (black) and after incubation with alkaline phosphatase (orange) at 4ºC. **b**. Cryo-EM data processing for KefC protein that was dialysed to remove AMP. The dataset contained 14,781 movies that were corrected by Patch motion correction and Patch CTF estimation in CryoSPARC[1]. After reference-

based auto-picking, 4,857,992 particles were picked. Several rounds of 2D classification were performed, yielding 66,197 particles, yet were unable to build a 3D reconstruction that had any clear map features for TM helices and the RCK domains were not visible.

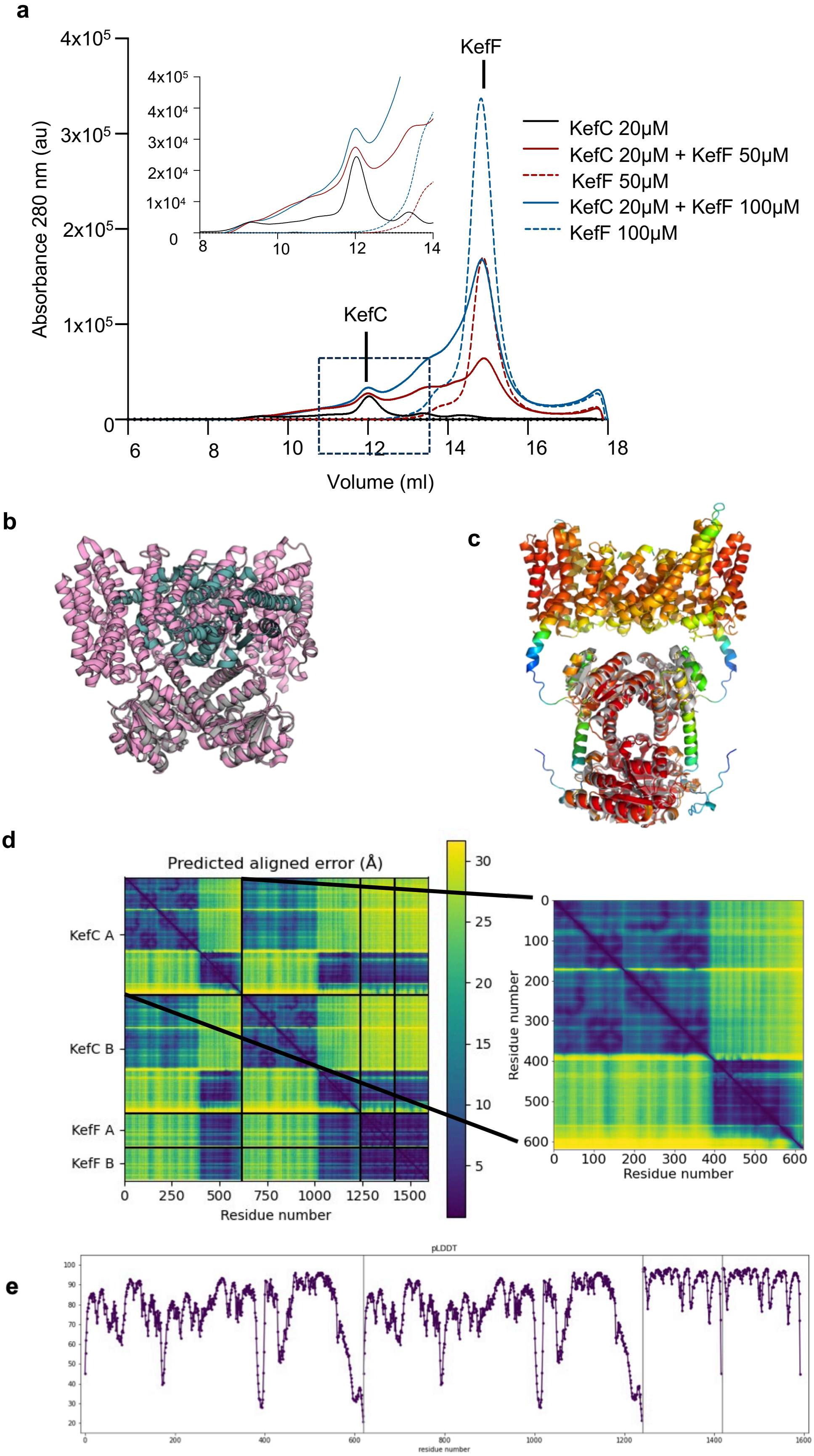
a
KefF
KefC
KefC 20μM
KefC 20μM + KefF 50μM
KefF 50μM
KefC 20μM + KefF 100μM
KefF 100μM
Absorbance 280 nm (au)
Volume (ml)
b
c
d
Predicted aligned error (Å)
KefC A
KefC B
KefF A
KefF B
Residue number
e
pLDDT
residue number

Supplementary Fig. 15. KefC and KefF interaction analysis. a. SEC profile of purified KefC (black) mixed with purified KefF (red and blue). Addition of KefF resulted in aggregation and no stable complex was observed. **b.** Superposition of cryo-EM structure of KefC WT* (pink) with RCK (Grey) + KefF (teal) complex (PDB: 3EYW) **c.** Complex containing KefC and KefF dimers as predicted by AlphaFold2[6]. The structure is coloured according to the pLDDT score associated to the corresponding amino acid, from lowest (blue, 20.73) to highest (red, 98.6). The previously crystallized RCK domain bound to KefF (PDB: 3EYW) is superimposed in light grey, showing good agreement with the predicted structure. **d.** Visualization of predicted aligned errors for a complex formed by KefC and KefF dimers as predicted by AlphaFold2[6]. The black lines separate the monomers. In the inset a magnification of a KefC monomer shows the transmembrane domain (highlighted in orange) and the RCK domain (highlighted in red). While intra-domain predicted aligned error are low (blue), the inter-domain shows a high error (green-yellow), indicating an ambivalence in their relative position and orientation. **e.** Visualization of predicted local-distance difference test (pLDDT) for a complex formed by KefC and KefF dimers as predicted by AlphaFold[6]. The monomers are separated by grey lines.

Name	Primer	Sequence
KefC WT*	Forward primer	GGGCGCGCCATATGGATAGCCATACGCTGATTCAGGCG
	Reverse primer	CGCGCGGGATCCAACCATTGCCATCTCTTCCACCATC
KefC -Δ 379	Forward primer	GGGCGCGCCATATGGATAGCCATACGCTGATTCAGGCG
	Reverse primer	CGCGGGATCCATTGAGGATCACCAGCAGAATCGGCGTTGC
KefC-Δ399	Forward primer	GGGCGCGCCATATGGATAGCCATACGCTGATTCAGGCG
	Reverse primer	CGCGGGATCCCTGTTCTTCGTCGATCTCATCGGCTTC
KefC D156N	Forward primer	GTGCTGCTGTTCCAGAATATCGCGGCGATCCCGC
	Reverse primer	GCGGGATCGCCGCGATATTCTGGAACAGCAGCAC
KefC Q155D	Forward primer	GTGCTGCTGTTCGACGATATCGCGGCGATCCCGC
	Reverse primer	GCGGGATCGCCGCGATATCGTCGAACAGCAGCAC
KefC K81A	Forward primer	CAAAGGCTGTGGGCGCTGCGTGCGGC
	Reverse primer	GCCGCACGCAGCGCCCACAGCCTTTG
KefC N135A	Forward primer	CATGCAGGCGATGGCTGAACGCAATCTGATG
	Reverse primer	CATCAGATTGCGTTCAGCCATCGCCTGCATG
KefC R146A	Forward primer	GACGCAAATGGGTGCCAGTGCCTTTGCG
	Reverse primer	CGCAAAGGCACTGGCACCCATTTGCGTC
KefC H259A	Forward primer	CGGAATACCGTGCTGCGCTGGAGAGCGAT
	Reverse primer	ATCGCTCTCCAGCGCAGCACGGTATTCCG
KefC K307A	Forward primer	GTTTCCTCATCATCGCAATCGCCATGCTG
	Reverse primer	CAGCATGGCGATTGCGATGATGAGGAAAC
KefC R401A	Forward primer	GAAGAACAGCCGGCAGTGATTATCGCC
	Reverse primer	GGCGATAATCACTGCCGGCTGTTCTTC
KefC E465A	Forward primer	GCGAAAGCGGCAGTGCTGATTAAC
	Reverse primer	GTTAATCAGCACTGCCGCTTTCGC
KefC R543A	Forward primer	GTATGAAGCGCGAGAAGCTGCCGATGTGTTC
	Reverse primer	GAACACATCGGCAGCTTCTCGCGCTTCATAC
KefC T127V	Forward primer	GCGCTCTCCTCTGTGGCGATTGCCATGCAGGCG
	Reverse primer	CGCCTGCATGGCAATCGCCACAGAGGAGAGCGC
KefF	Forward primer	AGGTCTAGAATGATTCTTATAATTTATGCGCATCCGTAT
	Reverse primer	ATACTCGAGACCATGATGGGCCTCCTGC

Supplementary Table 1. List of primers.

Supplementary References

1 Punjani, A., Rubinstein, J. L., Fleet, D. J. & Brubaker, M. A. cryoSPARC: algorithms for rapid unsupervised cryo-EM structure determination. *Nat Methods* **14**, 290-296 (2017).

2 The PyMOL Molecular Graphics System v. Version 1.2r3pre (Schrödinger, LLC).

3 Matsuoka, R. *et al.* Structure, mechanism and lipid-mediated remodeling of the mammalian Na(+)/H(+) exchanger NHA2. *Nat Struct Mol Biol* **29**, 108-120 (2022).

4 Okazaki, K. I. *et al.* Mechanism of the electroneutral sodium/proton antiporter PaNhaP from transition-path shooting. *Nat Commun* **10**, 1742 (2019).

5 Ashkenazy, H. *et al.* ConSurf 2016: an improved methodology to estimate and visualize evolutionary conservation in macromolecules. *Nucleic Acids Res* **44**, W344-350 (2016).

6 Jumper, J. *et al.* Highly accurate protein structure prediction with AlphaFold. *Nature* **596**, 583-589 (2021).
