# Supplementary material for: Structure and mechanism of the K+/H+ exchanger KefC
Source: Nat Commun. 2024 Jun 4;15:4751. doi: 10.1038/s41467-024-49082-7 (PMC11150392; doi:10.1038/s41467-024-49082-7)
Supplement: Supplementary file 3 — Description of Additional Supplementary Files [file 41467_2024_49082_MOESM3_ESM.pdf]

File name: Supplementary Movie 1
Description: Movie of the 3D Variability Analysis (3DVA) carried out in cryoSPARC on the cryo-EM map reconstruction of KefC WT* with AMP and GSH containing 397,824 particles processed in C1 symmetry.

File name: Supplementary Movie 2
Description: Movie of the 3D Variability Analysis (3DVA) carried out in cryoSPARC on the final cryo-EM map reconstruction of KefC with AMP containing 305,737 processed in C1 symmetry.
